# Production of lactic acid using a new homofermentative *Enterococcus faecalis* isolate

**DOI:** 10.1111/1751-7915.12133

**Published:** 2014-06-03

**Authors:** Mohan Raj Subramanian, Suvarna Talluri, Lew P Christopher

**Affiliations:** 1 Center for Bioprocessing Research and Development, South Dakota School of Mines and TechnologyRapid City, SD, 57701, USA; 2 Department of Civil and Environmental Engineering, South Dakota School of Mines and TechnologyRapid City, SD, 57701, USA

## Abstract

Lactic acid is an intermediate-volume specialty chemical for a wide range of food and industrial applications such as pharmaceuticals, cosmetics and chemical syntheses. Although lactic acid production has been well documented, improved production parameters that lead to reduced production costs are always of interest in industrial developments. In this study, we describe the production of lactic acid at high concentration, yield and volumetric productivity utilizing a novel homofermentative, facultative anaerobe *Enterococcus faecalis* CBRD01. The highest concentration of 182 g lactic acid l^−1^ was achieved after 38 h of fed-batch fermentation on glucose. The bacterial isolate utilized only 2–13% of carbon for its growth and energy metabolism, while 87–98% of carbon was converted to lactic acid at an overall volumetric productivity of 5 g l^−1^ h^−1^. At 13 h of fermentation, the volumetric productivity of lactate production reached 10.3 g l^−1^ h^−1^, which is the highest ever reported for microbial production of lactic acid. The lactic acid produced was of high purity as formation of other metabolites was less than 0.1%. The present investigation demonstrates a new opportunity for enhanced production of lactic acid with potential for reduced purification costs.

## Introduction

Lactic acid (C_3_H_6_O_3_, MW 90.08) is a chiral, three-carbon carboxylic acid recognized as GRAS (generally regarded as safe) by the US Food and Drug Administration because of its unique physicochemical properties and the diverse applications in food and other chemical industries (Narayanan *et al*., 2004). The world production of lactic acid in 2009 was 258 000 tons, with a projected growth of 329 000 tons by year 2015, and 367 000 tons by 2017 (Global Industry Analysts Inc, 2011). The estimated consumption of lactic acid in the US alone is 30 million pounds per year with a potential annual demand of 5.5 billion pounds, and the global consumption of lactic acid is expected to increase rapidly in the near future (Bajpai, 2013). This growth would mainly come from the lactic acid consumption in chemical applications. For example, the use of polylactic acid and ethyl lactate is expected to expand 19% per year (Jarvis, 2001; Garlotta, 2002; Södergård and Stolt, 2010). With a current price of $1.5/kg for a 88% purity food-grade product (www.icispricing.com), lactic acid has the potential to become a very large volume, commodity-chemical intermediate (Bozell and Petersen, 2010).

Lactic acid can be produced chemically using petrochemical feedstocks such as lactonitrile (Narayanan *et al*., 2004). However, the chemical synthesis produces a racemic mixture of D(−)-lactic acid and L(+)-lactic acid, which is not suitable for the synthesis of polylactic acid, as the optical purity of lactic acid is one of the major factors that determine the physical properties of its polymer. In contrast, about 90% of current commercial lactic acid is obtained via biological fermentation of sugars (Hofvendahl and Hahn-Hagerdal, 2000; Zhou *et al*., 2003). Microbial fermentation of sugars produces stereo-specific D(−)-lactic acid or L(+)-lactic acid depending on the strains used. For example, *Lactobacillus*, *Bacillus*, *Rhizopus*, *Streptococcus* and *Enterococcus* produce L(+)-lactic acid, while *Leuconostoc* and *La. vulgaricus* produce D(−)-lactic acid (Park *et al*., 2010). L(+)-lactic acid is the preferred component of many food and industrial applications that is currently produced via biological fermentation utilizing lactic acid bacteria or fungi such as *Rhizopus*. Some recombinant yeast strains have also been used to produce lactic acid from various carbon feedstocks (Porro *et al*., 2008). However, the yield and productivities of fungal and yeast strains are very low compared with lactic acid bacteria. In addition, the mycelial morphology of fungal strains affects the viscosity of the fermentation medium and can cause blockage during fermentation (Maas *et al*., 2006; Zhang *et al*., 2007; Ilmen *et al*., 2013). Therefore, these organisms require further gene manipulations and process development for the industrial production of lactic acid.

Recently, we have isolated a bacterial strain with high potential for lactic acid production. This strain was identified as *Enterococcus faecalis* and partially characterized. Subsequently, its full genome was sequenced, and the isolate *E. faecalis* CBRD01 was deposited with the American Type Culture Collection with a patent deposition designation PTA-12846 (unpublished results). Here we describe the abilities of *E. faecalis* CBRD01 to produce L(+)-lactic acid from glucose at high yields, titres and productivity with a minimum by-product formation at both flask and bioreactor scales.

## Results

### Morphology and biochemical characteristics of isolate CBRD01

*Enterococcus faecalis* CBRD01 belongs to Gram-positive elongated Cocci with cell size of approximately 1.2–1.5 μm. CBRD01 was able to grow anaerobically under dark conditions at temperature between 30 and 45°C, with a temperature optimum of 37°C. CBRD01 was able to utilize trehalose, lactose, ribose, saccharose, melizitose, cellobiose, mannose, mannitol, sorbitol and inositol (Table 1). The strain did not show oxidase, catalase, alkaline phosphatase, β-galactosidase, aminopeptidase and urease activities. The cellular fatty acid compositional analysis indicated that the strain CBRD01 had a match with *E. faecalis* (data not shown).

**Table 1:** Biochemical characterization of isolate CBRD01

Characteristics	Reaction
Shape	Cocci (elongated)
Size (in diameter)	1.2–1.5
Gram-reaction	+
Aminopeptidase activity	−
Potassium hydroxide test	−
Oxidase activity	−
Catalase activity	−
Acid from	
Glucose	+
Trehalose	+
Mannitol	+
Raffinose	−
Lactose	+
Ribose	+
Saccharose	+
Arabinose	−
Melibiose	−
Sorbitol	+
Melezitose	+
L-Rhamnose	−
Cellobiose	+
Mannose	+
Inositol	+
Aldehyde dehydrogenase activity	+
Urease activity	−
Voges Proskauer test	+
β-Galactosidase activity	−
Alkaline phosphatase activity	−
Growth at 45°C	+
Growth at 50°C	−
pH optimum	7.0

+, positive reaction; −, negative reaction.

### Production of lactic acid by *E. faecalis* CBRD01 at flask scale

In order to examine the production of lactic acid, the strain *E. faecalis* CBRD01 was cultured in 100 ml serum bottles with 50 ml of LA5 medium containing three different concentrations of glucose (28.79, 56.13 and 110.22 mM) at 37°C and 150 r.p.m. in an orbital incubator shaker under anaerobic condition. The cells were inoculated at 0.1 ± 0.01 Optical Density at 600 nm (OD600), and fermentation was continued for 12 h. The results revealed that a maximum yield of lactate was obtained at 110.22 mM glucose (Table 2). On average, 27–34 mM of glucose was consumed regardless of the initial concentrations of glucose. However, a reverse relationship was observed between the initial glucose concentration and glucose consumption after 12 h of incubation. The glucose consumption at lower starting concentrations (28.79 and 56.13 mM) was 93% and 60% respectively. This was 3.3- and 2.1-fold, respectively, higher than that at 100.22 mM glucose (28%). The lactate concentration varied from 49 to 61 mmol l^−1^. In theory, 1 mol of glucose yields a maximum of 2 mol of lactate under anaerobic condition. Accordingly, the glucose consumption at 110.22 mM glucose concentration was 30.55 mM. The lactate titre was 60.21 mmol l^−1^ after 12 h, thus yielding 1.97 mol mol^−1^ glucose, which is equivalent to 98.54% of the theoretical maximum. However, it should be noted that lactate production ceased after 12 h, although the experiment was carried out for 24 h.

**Table 2:** Lactate production from different glucose concentrations by *E. faecalis* CBRD01 under anaerobic batch fermentation

Starting glucose concentration (mM)	Maximum specific growth rate *μ*_max_ (h^−1^)^a^	Glucose consumed (mM)^b^	Glucose uptake rate (mmol g^−1^ cdw h^−1^)^b^	Lactate produced^c^ (mmol l^−1^)^b^	Lactate production rate (mmol g^−1^ cdw h^−1^)^b^	Lactate yield (mol mol^−1^ glucose)^b^	Lactate yield (%)^b^
28.79	0.59	26.87	19.92	49.12	36.42	1.83	91.39
56.13	0.59	33.91	23.89	60.84	42.86	1.79	89.71
110.22	0.64	30.55	21.22	60.21	41.83	1.97	98.54

a Estimated between 0 and 3 h.

b Estimated for 12 h.

c Optically pure L(+)-lactic acid.

### Production of lactic acid by *E. faecalis* CBRD01 in batch process at bioreactor scale

**Figure 1:**
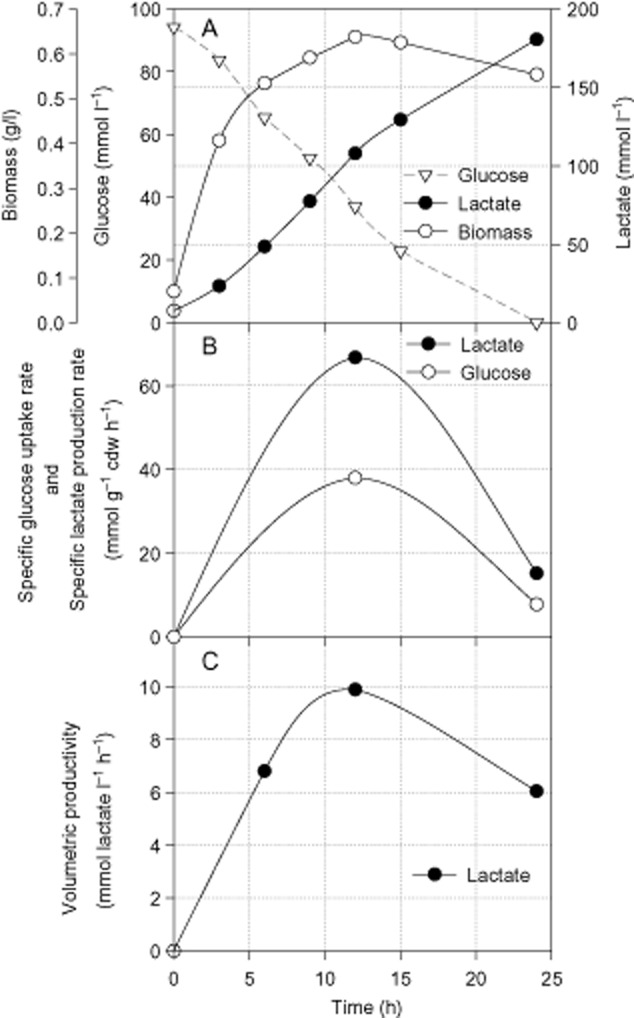
Time-course profile of growth, glucose consumption and lactate production by *E. faecalis* CBRD01 under anaerobic batch fermentation.A. Glucose consumption, biomass and lactate production.B. Specific rates of glucose uptake and lactate production.C. Volumetric productivity of lactate.

To investigate the potential of *E. faecalis* CBRD01 for lactic acid production at bioreactor scale, the strain was cultured under controlled conditions at a constant pH of 7.0 ± 0.2 in a 1 l glass jar bioreactor (DASGIP) with a working volume of 0.5 l for 24 h. Results are shown in Fig. 1. The initial glucose concentration was 94 mM. In 24 h of the bioreactor culture, *E. faecalis* CBRD01 produced 172.8 mmol lactate l^−1^, which is 2.87-fold higher than the titre obtained from the flask culture. The biomass production reached a maximum of 0.64 g l^−1^ at 12 h (from 0.07 g l^−1^ at 0 h) and then decreased to 0.62 g l^−1^ at 24 h (Fig. 1A). Both the specific glucose uptake rate and specific lactate production rate peaked at 12 h of cultivation (Fig. 1B), with the former being 0.57% of the latter. At 12 h, a maximum volumetric productivity of 10 mmol^−1^ l^−1^ h^−1^ was attained (Fig. 1C). Therefore, to better understand the behaviour of CBRD01, it was of interest to discuss its fermentation profile in two phases (phase 1, 0–12 h; phase 2, 12–24 h).

The total glucose consumption at 24 h was 94.06 mmol l^−1^ (Table 3). Although the lactate yield in the first half of cultivation (0–12 h) was 88%, it improved to 98% in the second (12–24 h), resulting in an overall yield of 91.86 % of theoretical maximum of glucose. The *μ*_max_, estimated between 0 and 3 h, was 0.59 h^−1^. The maximum specific production rate (*q*_max_) of lactate, estimated between 0 and 12 h (phase 1), was 66.72 mmol g cdw^−1^ h^−1^. In phase 2 (12–24 h), *q*_max_ decreased to 15 mmol g cdw^−1^ h^−1^.

**Table 3:** *Enterococcus faecalis* CBRD01 fermentative characteristics of lactate production from glucose under anaerobic batch fermentation

Biomass and lactate production	Cultivation time		
	0–12 h	12–24 h	0–24 h
Biomass (g l^−1^)	0.57	0.00	0.57
Glucose utilized (mmol l^−1^)	57.04	37.02	94.06
Lactate produced (mmol l^−1^)^a^	100.25	72.55	172.8
Maximum specific growth rate (h^−1^)^b^	0.59	0.00	−
Biomass yield (g cdw^c^ g^−1^ glucose)	0.06	0.00	0.03
Specific glucose uptake rate (mmol g^−1^ cdw h^−1^)	37.96	7.75	32.53
Specific lactate production rate (mmol g^−1^ cdw h^−1^)	66.72	15.20	59.76
Lactate yield (mol mol^−1^ glucose)	1.75	1.96	1.84
Lactate yield (g g^−1^ glucose)	0.88	0.98	0.92
Lactate final yield (% of theoretical)^d^			91.86

a Optically pure L(+)-lactic acid.

b Calculated between 0 and 3 h.

c cdw, cell dry weight.

d Accounted for 0–24 h.

**Figure 2:**
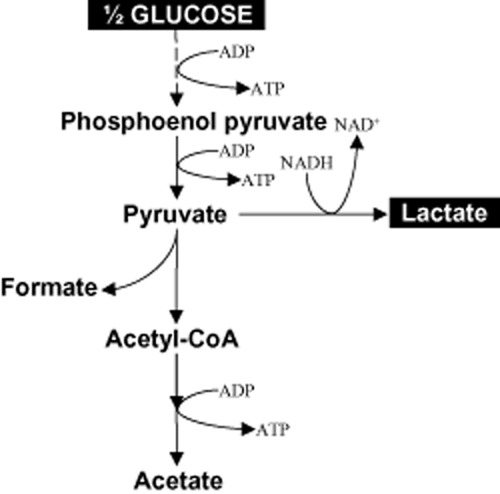
Proposed metabolic pathway for lactate production from glucose in *E. faecalis* CBRD01.

Lactate was the major product of the fermentation process with *E. faecalis* CBRD01. Other metabolites, such as acetate and formate, were produced in quantities of less than 4% in the 1st phase and less than 0.6% in the 2nd phase (Table 4). Analysis of the carbon material balance revealed that 101.16% of glucose carbon was recovered in phase 1 (0–12 h) and 98.9% in phase 2 (12–24 h). In phase 1, 87.9% of glucose carbon was converted as lactate, while 6.2% of carbon was directed towards biomass formation. However, no carbon was utilized for biomass in the second phase, while 0.6% of carbon was utilized for acetate and 0.3% for formate, thus yielding 98% of lactate in the second phase. The formate and acetate co-metabolites were produced in very low quantities of less than 1 g l^−1^. The electron balance, calculated for the substrates and products, indicated 0.99–1.0 (Table 4). This means that the electrons, released during glucose oxidation, were completely recovered in the form of products. Based on the carbon distribution of glucose into metabolites (no metabolites were omitted during analysis), a carbon metabolic pathway for *E. faecalis* CBRD01 is proposed in Fig. 2, suggesting that this strain utilizes a homolactic fermentative pathway to produce lactic acid.

**Table 4:** Carbon material balance of glucose metabolism by *E. faecalis* CBRD01 under anaerobic batch fermentation

Substrate and metabolites^a^	Molecular formula	Cultivation time									
		Phase I (0–12 h)					Phase II (12–24 h)				
		mM	C-mM	C (%)	γ_i_^b^	γ_i_ balance (mol l^−1^)^c^	mM	C-mM	C (%)	γ_i_^b^	γ_i_ balance (mol l^−1^)^c^
Substrate											
Glucose	C_6_H_12_O_6_	57.04	342.24	100.00	4	1.37	37.02	222.12	100.00	4	0.89
Biomass	CH_1.8_ O_0.5_ N_0.2_	0.52^d^	21.22^e^	6.20	4.8	0.10	0.00^d^	0.00	0.00^e^	4.8	0.00
Metabolites											
Lactate^f^	C_3_H_6_O_3_	100.25	300.75	87.88	4	1.20	72.55	217.65	97.99	4	0.87
Acetate	C_2_H_4_O_2_	6.51	13.01	3.80	4	0.05	0.65	1.30	0.59	4	0.01
Formate	CH_2_O_2_	11.22	11.22	3.28	2	0.02	0.67	0.67	0.30	2	0.00
Carbon dioxide	CO_2_	0.00	0.00	0.00		0.00	0.00	0.00	0.00		0.00
Total products			346.20			1.38		1350.13			0.88
Reduction deg. balance						1.00					0.99
Carbon recovery %^g^				101.16					98.88		

a The standard deviation of the measurements was less than 3% for the substrate and the metabolites.

b Degree of reduction (γ_i_), which was calculated using the formula: γ_i_ = (4*a* + *b* − 2*c* + 6*e* + 5*f*)/*a*, where *a* denotes carbon, *b* denotes hydrogen, *c* denotes oxygen, *e* denotes sulfur, and *f* denotes phosphorus (Gustafsson *et al*. 1993).

c Degree of reduction (γ_i_) balance, which was calculated as γ_i_ for total products/γ_i_ for substrate (expressed in mol l^−1^).

d g l^−1^_._

e An average molecular weight of 24.6, which corresponds to an average cell with a molecular formula of CH_1.8_ O_0.5_ N_0.2_. The average ash content of 8% was deduced from the actual cell dry mass (Stephanopoulos *et al*., 1998).

f Optically pure L(+)-lactic acid.

g Total carbon in biomass and metabolites, Total carbon in glucose^−1^ × 100.

### Production of lactic acid by *E. faecalis* CBRD01 in fed-batch process at bioreactor scale

**Figure 3:**
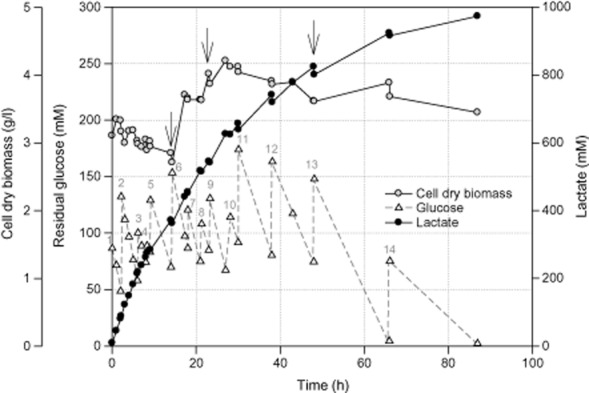
Time-course profile of growth, glucose consumption and lactate production by *E. faecalis* CBRD01 under anaerobic fed-batch fermentation. Arrows indicate addition of yeast extract at 5 g l^−1^. The numbers 1–14 in grey indicate glucose addition at different time intervals and different concentrations (1–86.4 mM; 2–83.74 mM; 3–42.07 mM; 4–14.63 mM; 5–46.04 mM; 6–83.59 mM; 7–33.82 mM; 8–33.17 mM; 9–45.71 mM; 10–46.98 mM; 11–82.34 mM; 12–82.80 mM; 13–73.24 mM; and 14–70.75 mM).

To further evaluate the potential of *E. faecalis* CBRD01 for lactic acid production, a fed-batch process was carried out in a 1 l glass jar bioreactor with a 0.5 l working volume of LA5 medium at pH 7.0 and 37°C for 87 h under anaerobic conditions. The initial glucose concentration was 86.4 mM. During the fermentation period, a total of 825 mM of glucose was added in 14 batches (Fig. 3). The fed-batch process was initiated by adding 3.0 g l^−1^ of *E. faecalis* CBRD01 cells, grown under anaerobic conditions, to the culture medium. The total glucose consumption at the end of the fermentation process was 823 mmol l^−1^. The strain produced 973 mmol l^−1^ lactate in 87 h. This translates into a lactate yield of 59% from glucose, with an average productivity of 1.0 g l^−1^ h^−1^ (Fig. 3). However, the lactate yield in the first phase (0–12 h) was above 91%. When compared with the batch production of lactic acid after 24 h (Fig. 1), the fed-batch process increased lactic acid production approximately threefold (532 mM).

### Fed-batch bioreactor production of lactic acid by *E. faecalis* CBRD01at high cell density culture

**Figure 4:**
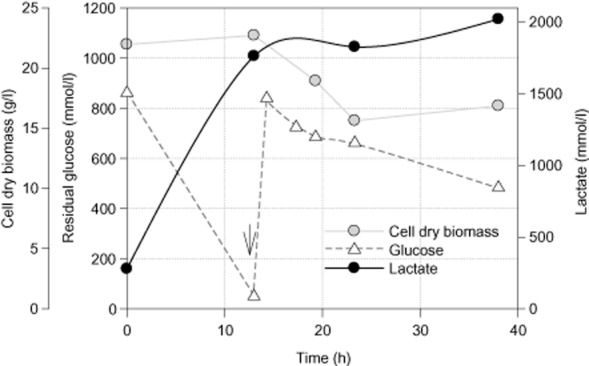
Time-course profile of growth, glucose consumption and lactate production by *E. faecalis* CBRD01under anaerobic fed-batch fermentation with initial cell density of 22 g l^−1^. Arrow indicates glucose addition.

In an attempt to increase the lactic acid titres, fed-batch experiments were performed using a high cell density culture (22 g l^−1^) and LA6 culture medium in a 1 l glass jar bioreactor (0.3 l working volume). Lactic acid production was carried out under anaerobic conditions at pH 7.0 and 37°C for 38 h (Fig. 4). At the end of the fermentation process, *E. faecalis* CBRD01, grown in LA6 medium, was able to produce 2022 mmol lactate l^−1^, which is equivalent to 182.1 g l^−1^. The total glucose consumption was 210.01 g with an overall lactate yield of 86.7% from the theoretical maximum on glucose. The microbial biomass, retained at the end of fermentation, was 16.86 g cdw l^−1^. About 811 mM of glucose was consumed in 13 h (before glucose addition) to produce 1484 mmol lactic acid l^−1^, which represents a 91.5% yield of the theoretical maximum on glucose or 1.83 mol lactate mol^−1^ glucose. The volumetric productivity accounted between 0 and 13 h was 114 mmol^−1^ l^−1^ h^−1^ (10.3 g l^−1^ h^−1^), which is the highest volumetric productivity ever reported for microbial production of lactic acid. As the energy requirement for maintaining high cell density is very high, there was no net cell growth. The biomass production remained unchanged for 13 h and thereafter declined due to the dilution effect that occurred with the glucose spiking.

## Discussion

The production of lactic acid for commercial applications requires the use of less fastidious, but robust microorganisms that can meet the industrial needs for high titre, high productivity and high yield of lactate of at least 100 g l^−1^, 2.5 g l^−1^ h^−1^ and 80% respectively (Werpy and Petersen, 2004). As purification costs comprise a significant portion of the overall production costs, the purity of food grade (80–90% purity) and pharmaceutical grade (> 90%), lactic acid determines its price (John *et al*., 2007). Therefore, to minimize the production and purification costs, there is a need of new microbial isolates with enhanced capabilities for high-purity lactic acid production.

Here, we describe a wild-type bacterial strain, recently isolated and identified as *E. faecalis* CBRD01. This strain is different from other known lactic-producing *Enterococcus* sp., such as *E. asini*, *E. durans*, *E. hirae*, *dispar*, *E. facium*, *E. solitaries*, *E. malodoratus*, *E. pseudoavium* and *E. saccharolyticus* in that it utilizes mannitol, sorbitol, ribose and melezitose as carbon sources for lactic acid production. However, while *E. faecalis* CBRD01 does not produce acid from arabinose, xylose, melibiose and raffinose, most of the other *Enterococcus* sp. such as *E. avium*, *E. casseliflavus*, *E. gallinarum*, *E. mundtii*, *E. cecorum*, *E. raffinosus* and *E. saccharolyticus* can ferment the previous carbon sources to lactic acid (Manero and Blanch, 1999).

*Enterococcus faecalis* CBRD01was able to produce high yields and titres of lactic acid with minimal impurities on glucose. The response of CBRD01 to different glucose concentrations is of particular interest because of the direct impact of parameters such as substrate inhibition, viscosity of culture medium, by-product formation on the cellular metabolisms and product formation (Booth, 1985; Hall *et al*., 1995). Initial glucose concentrations of up to 110 mM had no adverse effect on cell growth of CBRD01 as *μ*_max_ (estimated between 0 and 3 h) remained constant (0.59 h^−1^) at 29 and 56 mM glucose, and slightly increased (0.64 h^−1^) at 110 mM glucose. Acetate and formate were the only co-metabolites detected at concentrations of less than 1 mM, suggesting that the formation of lactate by-products and their inhibitory role on glucose fermentation was negligible. However, the increase in the initial glucose concentrations from 29 to 110 mM did not improve glucose consumption. In fact, at 110 mM glucose, the total glucose consumption was lower than at 56 mM glucose. Furthermore, the rate of glucose uptake did not change substantially and remained in the range of 19.9–23.9 mmol g^−1^ cdw h^−1^ at all three glucose concentrations. This suggests that (i) the CBRD01 cells were not under stress at high glucose concentrations (110 mM), and (ii) the cellular metabolism of lactate production was not influenced by the glucose concentration. The latter was evident from the lactate yield that was in the range of 90–99% at all three glucose concentrations.

While examining the pH of the culture broth after 12 h of fermentation, it was noticed that pH decreased to 5.3 ± 0.3 from the initial pH 7.0, regardless of the initial glucose concentrations. The pH drop, which was due to the formation of lactic acid, could be responsible for the cease of further biomass and lactate production in *E. faecalis* CBRD01. In order to understand the effect of pH on improving lactate production, a batch process was conducted under pH-controlled conditions in a 1 l glass jar bioreactor. When the pH of the culture medium was maintained at 7.0 ± 0.2 throughout fermentation, 173 mmol lactate l^−1^ was produced from 94 mmol glucose l^−1^, which is equivalent to a 92% lactate yield on glucose carbon. Hence, the culture pH had a major impact on glucose metabolism and lactate production.

Although the batch process showed a 2.9-fold improvement in lactate production compared with shake flask experimental results, the supply of initial glucose was seen as a limiting factor for increased lactate production. Therefore, a fed-batch process was designed to better understand the lactate production potential of *E. faecalis* CBRD01. In a separate study, the effect of inoculum load was examined (data not shown). It was observed that at a given lactate titre, the use of higher inoculum densities (on dry weight) reduced the fermentation time to reach that titre. Alternatively, a higher lactate titre can be attained at the same fermentation time. For example, a fed-batch process with 3 g l^−1^ inoculum increased lactate production to 973 mmol l^−1^ from 823 mM of glucose in 87 h. When accounted at 24 h, the lactate titre was 541 mmol l^−1^, and the total glucose consumed was estimated at 338 mmol l^−1^. This corresponds to a lactate yield of 80% on glucose, which is threefold higher than the batch production of lactate after 24 h. However, at 13 h of fed-batch fermentation, the biomass concentration declined to 2.85 g l^−1^ from 3.0 g l^−1^. Although the culture medium was maintained at glucose concentrations in excess of 60 mM, the carbon to cell growth and biomass maintenance with CBRD01 were low. This necessitated addition of complex nitrogen sources such as yeast extract to maintain biomass concentration.

To further improve the lactate production in *E. faecalis* CBRD01, fermentation parameters including medium composition, initial glucose concentrations and inoculum density were varied and modified. CBRD01 growth and lactate production were examined as a function of these parameters (data not shown). Results suggested the possibility of improving lactate production by using a high cell density culture of 22 g l^−1^ cells and a high glucose concentration of up to 1 M on an altered medium LA6. Under those conditions, CBRD01 produced 2.02 mol lactate l^−1^ (equivalent to 182 g lactate l^−1^) at an overall lactate yield of 87% on glucose carbon. The overall volumetric rate of lactate production was estimated to be 56 mmol l^−1^ h^−1^ (equivalent to 5 g lactate l^−1^ h^−1^). A similar strain, *E. faecalis* RKY1 was previously reported to ferment glucose at an optimal concentration of 150 g l^−1^ to lactic acid with a final titre of 144 g l^−1^, at an optimal productivity of 5.1 g l^−1^ h^−1^ (Yun *et al*., 2003; Wee *et al*., 2004; 2006a,b). In comparison, the present investigation demonstrates a higher lactate titre at a similar volumetric productivity.

Recently, two *Bacillus* strains were used to produce lactic acid (Ou *et al*., 2011; Meng *et al*., 2012). High lactic acid titres were obtained on glucose and xylose. For example, a thermotolerant *B. coagulans* 36D1 strain produced 182 g l^−1^ of lactic acid with a carbon yield of 92.3% on glucose following a fed-batch fermentation for 261 h. The same strain could also produce lactic acid at 163 g l^−1^ from 86.3 g l^−1^ of xylose, thus yielding 87.3% on xylose carbon equivalents. However, the volumetric productivities of lactic acid production estimated from the fed-batch process with both glucose and xylose were less than 0.84 g l^−1^ h^−1^ (Ou *et al*., 2011). In another report (Meng *et al*., 2012), an alkaliphilic *Bacillus* sp. WL-S20 was shown to produce a maximum of 225 g l^−1^ of lactic acid in a fed-batch process at a productivity rate of 1.04 g l^−1^ h^−1^, while a single-pulse feeding of glucose yielded a maximum of 180 g l^−1^ of lactic acid at 1.61 g l^−1^ h^−1^ after 112 h of fermentation. Both *Bacillus* strains were very efficient in producing lactic acid at high concentrations, comparable or exceeding those of *E. faecalis* CBRD01, but at threefold to fivefold lower volumetric productivity rates.

*Enterococcus faecalis* CBRD01, believed to use a homolactic fermentative pathway to produce L(+)-lactic acid, utilized 2–13% of carbon for its growth and energy metabolism, with the rest of the carbon converted to lactic acid at an overall volumetric productivity of 5 g l^−1^ h^−1^. Formation of other metabolites such as acetate and formate was less than 0.1%. The fermentation characteristics and lactic acid-producing capabilities of CBRD01, as presented in this work, can certainly compete with existing published literature. Following further optimization, *E. faecalis* CBRD01 may become a strong candidate for industrial production of lactic acid.

## Experimental procedures

### Materials

Yeast extract (Cat. 212750) and tryptone (Cat. 211705) were purchased from Difco (Becton Dickinson, Franklin Lakes, NJ, USA). Glucose, lactic acid and all other chemicals and reagents, unless otherwise indicated, were purchased from Sigma-Aldrich (St. Louis, MO, USA).

### Culture enrichment, isolation and identification of isolate CBRD01

Soil samples, collected from the Material Recovery Facility in Rapid City, SD, USA, were placed in 100 ml sterile serum bottles containing 50 ml culture enrichment medium and tightly sealed using butyl rubber stoppers with aluminium caps. Before inoculating the samples, the serum bottles were flushed with nitrogen gas (99.99%) for 15 min to ensure that the bottles were completely deprived of oxygen. The samples were then processed for culture enrichment for lactic acid production. The culture enrichment medium for lactic acid production had the following components per litre of deionized water: NH_4_Cl, 1.6 g; MgSO_4_·7H_2_O, 0.25 g; NaCl, 1.0 g; FeCl_3_·6 H_2_O, 2.5 mg; Na_2_S_2_O_3_·5 H_2_O, 1.0 g; yeast extract, 5.0 g; tryptone, 5.0 g; glucose, 100 mmol (18.0 g); Pfennig and Lippert's trace element solution (PL 9), 1.0 ml. The medium was supplemented with 100 mM potassium phosphate buffer at pH 7.0. The Pfennig and Lippert's trace element solution contained the following components per litre of 77 mM HCl: FeSO4·7H_2_O, 0.21 g; ZnSO_4_·7H_2_O, 50 mg; MnCl_2_·4H_2_O, 50 mg; H_3_BO_3_, 15 mg; CoCl_2_·6H_2_O, 100 mg; CuSO_4_·5H_2_O, 6.7 mg; NiCl_2_·6H_2_O, 10 mg; Na_2_SeO_3_, 5.0 mg; Na_2_MoO_4_·2H_2_O, 15 mg. Enrichment cultures, displaying positive growth with high lactic acid titre after three consecutive transfers in the selective medium, were chosen for isolation.

A 0.1 ml of lactate-producing microbial culture was placed onto agar plates, containing selective medium, and incubated under anaerobic condition. In total, 55 different microbial strains were isolated from the soil samples (data not shown). Cultures that displayed rapid growth on glucose with lactic acid as the primary metabolite were resorted for further isolation and verified for purity by streaking on medium solidified with agar. The most promising isolate with high lactate-producing capability on glucose was referred to as CBRD01 and used in this study. The strain CBRD01 was biochemically characterized (Table 1) and identified as *E. faecalis* by DSMZ-Germany (http://www.dsmz.de/). This strain was referred to as *E. faecalis* CBRD01.

### Flask cultivation of *E. faecalis* CBRD01

To examine the production of lactic acid, the strain *E. faecalis* CBRD01 was cultured in 100 ml serum bottles with 50 ml working volume in an orbital incubator shaker (Innova R42, Eppendorf, USA) at 150 r.p.m. under anaerobic condition. The lactic acid medium (LA5 medium) contained the following components per litre of deionized water: NH_4_Cl, 1.6 g; MgSO_4_·7H_2_O, 0.25 g; NaCl, 1.0 g; FeCl_3_·6 H_2_O, 2.5 mg; Na_2_S_2_O_3_·5 H_2_O, 1.0 g; yeast extract, 3.0 g; Pfennig and Lippert's trace element solution (PL 9), 1.0 ml. The pH of the culture medium was adjusted to pH 7.0 with 100 mM potassium phosphate buffer. Three different concentrations of glucose, 25 mM (4.5 g l^−1^), 50 mM (9 g l^−1^) and 100 mM (18 g l^−1^), were used in the fermentation studies. The cells were inoculated at 0.1 ± 0.01 OD_600_, and fermentation was carried out at 37°C for 12 h. Samples were withdrawn periodically to determine cell mass, residual glucose and metabolites.

### Bioreactor cultivation of *E. faecalis* CBRD01

A 1 l glass jar bioreactor (DASGIP-FB04CS, Eppendorf, Germany) with a working volume of 500 ml of LA5 medium containing 100 mM potassium phosphate buffer (pH 7.0) was used. Glucose was added at 86.4 mM. The dissolved oxygen was expelled by flushing the reactor with nitrogen gas (99.99%) for 30 min at 1.0 vol vol^−1^ min^−1^. The strain was grown overnight and then inoculated at an OD_600_ of 0.2 into the medium. The temperature and agitation rate were maintained at 37°C and 250 r.p.m. respectively. The pH was maintained at 7.0 ± 0.1 with 2.5 N NaOH and 2.5 N HCl. Samples were withdrawn periodically to determine the biomass, residual substrate and metabolite concentration. Fermentation was continued for 72 h. For fed-batch fermentation, concentrated glucose (4.0 M) was added intermittently. The glucose content in the medium was monitored periodically using high-performance liquid chromatography (HPLC), and its concentration was maintained above 60 mM until 60 h.

An improved fed-batch process was carried out at 37°C in a 1 l glass jar bioreactor with a 0.3 l working volume at high cell density using LA6 medium, which contained the following components per litre of deionized water: yeast extract, 18 g; tryptone, 9.0 g; dipotassium phosphate, 9.38 g; monopotassium phosphate, 1.73 g; Lippert's trace element solution, 1.0 ml. After sterilization, the fermenters were flushed with N_2_ gas for 1 h to ensure oxygen-free environment. Fermentation was initiated by adding *E. faecalis* CBRD01 cells, grown under anaerobic conditions, at 22 g l^−1^. Glucose was added at 0.86 M to the fermenter. The pH of LA6 was maintained at pH 7.0 ± 0.2 using 10 N NaOH and 5.0 N HCl solution throughout fermentation. Experiments were conducted for 38 h.

### Analytical methods

Cell concentrations were measured in a 10 mm path length cuvette using a UV-2450 double-beam spectrophotometer (Shimadzu, Kyoto, Japan) at 600 nm. One unit of absorbance at 600 nm corresponded to 0.3 g cell dry weight (cdw) l^−1^. Concentrations of glucose and fermentation metabolites (lactate, acetate, formate) were determined by the method described previously using Shimadzu LC20 HPLC (Shimadzu) equipped with a refractive index detector (model−RID-10A, Shimadzu) (Talluri *et al*., 2013). Briefly, the supernatants, obtained by centrifugation of the culture samples at 10 000 × *g* for 10 min, were filtered through the Nylon-membrane (Cole-Parmer, Vernon Hills, IL, USA) and eluted through a 300 × 7.8 mm Aminex HPX-87H (Bio-Rad, Hercules, CA, USA) column at 60^o^C using 5.0 mM H_2_SO_4_.

### Calculation of fermentation parameters

The maximum specific growth rate (*μ*_max_; h^−1^) was estimated by plotting the total cell concentration against time in a log-linear plot. The slope of the straight line during exponential growth was used to find the average specific growth rate. Specific rates (glucose uptake rate or lactate production rate) were calculated by dividing the volumetric rates by the time average concentration of cells (mmol g cdw^−1^ h^−1^). Consumption of glucose and production of lactate were used to calculate the volumetric rates or productivities (mmol l^−1^ h^−1^). Lactate yield on glucose (mol mol^−1^) was estimated by dividing lactate concentration (mol) by glucose consumption (mol). Biomass yield (g cdw g glucose^−1^) was estimated from the cell mass produced (on cell dry weight) per gram of glucose consumed.

## Conflict of interest

None declared.
